# Alcohol Hangover Across the Lifespan: Impact Of Sex and Age

**DOI:** 10.1093/alcalc/agab027

**Published:** 2021-04-05

**Authors:** Joris C Verster, Noortje R Severeijns, Annabel S M Sips, Hama M Saeed, Sarah Benson, Andrew Scholey, Gillian Bruce

**Affiliations:** 1 Division of Pharmacology, Utrecht Institute for Pharmaceutical Sciences (UIPS), Utrecht University, Utrecht 3584 CG, The Netherlands; 2 Centre for Human Psychopharmacology, Swinburne University of Technology, Melbourne VIC 3122, Australia; 3 Division of Psychology and Social Work, School of Education and Social Sciences, University of the West of Scotland, Paisley PA1 2BE, UK

## Abstract

**Aim:**

To investigate the relationship between age and hangover frequency and severity.

**Method:**

An online survey, generated through Facebook, collected self-report data relating to alcohol consumption from 761 Dutch alcohol consumers aged 18–94 years (61.6% female).

**Results:**

Overall, young individuals consumed more alcohol than older drinkers, and men more than women. Significant interactions between age group and sex were found for both subjective intoxication and hangover severity, indicating that the sex differences in these variables were greatest in the younger age groups but became significantly smaller or absent in the older age groups. Partial correlations, correcting for estimated blood alcohol concentration (eBAC), revealed significant and negative partial correlations between age and subjective intoxication (*r* = −0.444, *P* < 0.0001), age and hangover severity (*r* = −0.327, *P* < 0.0001) and between age and hangover frequency (*r* = −0.195, *P* < 0.0001), i.e. subjective intoxication, hangover severity and hangover frequency decline with age. With regard to sex differences, the observed correlations with age for the past month heaviest drinking occasion were stronger in men for subjective intoxication, (*z* = −2.25, *P* = 0.024), hangover severity (*z* = −3.36, *P* = 0.0008) and hangover frequency (*z* = −3.63, *P* = 0.0003).

**Conclusions:**

Hangover severity declines with age, even after controlling for eBAC or the amount of alcohol consumed. Sex differences were greatest in the younger age groups but became significantly smaller or absent in the older age groups. The relationship between age and hangover severity is strongly mediated by subjective intoxication. Pain sensitivity, lower with aging, might be a mediator.

## Teaser

**Short Summary:** This study shows that subjective intoxication, hangover severity and hangover frequency decline with age. The relationship between age and hangover severity is strongly mediated by subjective intoxication. An age-related decline in sensitivity to pain may in part explain the observed negative relationships.

## INTRODUCTION

The hangover is the most commonly reported negative consequence of alcohol consumption (Verster *et al.*, 2009) and has been defined as the combination of negative mental and physical symptoms which can be experienced after a single episode of alcohol consumption, starting when the blood alcohol concentration (BAC) approaches zero (Van Schrojenstein Lantman *et al.*, 2016; Verster *et al.,* 2020b). Alcohol consumption and the resulting hangovers typically start during teenage years and can continue into late life. Previous publications have suggested that research on age as an explanatory factor for hangovers is needed (Prat *et al.*, 2009; Verster *et al.*, 2010). However, a search of the literature since then shows that hangover research in age groups other than young adults (18–30 years old) is limited. As such, relatively little is known about whether older drinkers experience hangovers differently than younger drinkers.

When examining the literature, it is important to distinguish between hangover frequency and hangover severity. To date, only two studies have reported on the relationship between age and hangover frequency. In a prospective study, Piasecki *et al.* (2005) followed student drinkers for 11 years and found a steady decline in hangover frequency. Evaluating data from the nationally representative US National Health and Nutrition Examination Survey sample of 18–65–year-old participants, Tolstrup *et al.* (2014) also found that with increasing age, the frequency of hangovers gradually decreased. The observation by Tolstrup *et al.* (2014) that the occurrence of hangovers declined with increasing age remained after correction for the participants’ usual amount of alcohol intake and frequency of binge drinking. While the title of the paper suggests otherwise (‘Does the severity of hangovers decline with age? Survey of the incidence of hangover in different age groups’). Note that hangover frequency, rather than severity, was assessed in this study.

To the authors’ knowledge, to date, no studies have investigated whether hangover severity is stable across the lifespan. Theoretically repeated alcohol exposure during aging could lead to either tolerance or reverse tolerance to hangover (Verster *et al.*, 2019). Therefore, the purpose of the current study was to investigate the relationship between age and both frequency and severity of hangover. This was accomplished by evaluating data from drinkers’ heaviest drinking occasion during a 2-month period.

Two other factors were considered. First, the amount of alcohol consumed per drinking occasion declines with age (Tolstrup *et al.*, 2014). Second, subjective intoxication is one of the important predictors of hangover severity (Verster *et al.*, 2020a). Therefore, the possible impact of both alcohol intake and perceived intoxication were factored into the analysis of the relationship between age and hangover severity.

## METHODS

A subsample of alcohol consumers from an online survey was used for the current analysis (Kiani *et al.*, 2021). This online survey was conducted between 24 June and 26 July 2020, and data were collected on immune fitness and the psychosocial and health consequences of the COVID-19 pandemic lockdown in the Netherlands. The data used for the current analysis comprised alcohol consumption data for the period from 15 January to 14 March 2020 (i.e. the period prior to the COVID-19 lockdown). Participants who were 18 years and older and were invited via Facebook to complete the online survey. The study was conducted by Utrecht University, and the Ethics Committee of the Faculty of Social and Behavioral Sciences of Utrecht University granted ethical approval (approval code FETC17-061). Electronic informed consent was obtained from all participants before starting the survey.

The data analyzed comprised demographic information which included age, sex, weight and height. Questions about alcohol consumption were answered for the 2-month period before the COVID-19 lockdown (15 January–14 March 2020). During this period, there were no restrictions on normal bar and club openings that could have influenced the study outcome. Participants reported the number of alcoholic drinks they consumed on average per week and the number of days per week they consumed alcohol. Guidance was provided regarding serving sizes (e.g. glass and bottle) and how to convert these into units of alcohol.

With regard to the heaviest drinking occasion within the 2-month period, the number of alcoholic drinks consumed as well as the duration of drinking (hours) was reported. The estimated blood alcohol concentration (eBAC) for this occasion was computed using an adapted Widmark equation (Watson *et al.*, 1981) and by taking into account sex and body weight. Subjective intoxication (drunkenness) for the heaviest drinking occasion was rated on an 11-point scale ranging from 0 (totally not) to 10 (extremely drunk) (Van de Loo *et al.*, 2016). Using a similar scale, next-day hangover severity was assessed with a range from 0 (no hangover) to 10 (extremely severe hangover) (Verster *et al.*, 2020c). Finally, participants reported how many hangovers they had experienced in the 2-month period. By dividing this number by 2, the monthly number of experienced hangovers was computed.

Statistical analyses were conducted with SPSS (IBM Corp. Released 2013. IBM SPSS Statistics for Windows, Version 25.0. Armonk, NY: IBM Corp.). Mean and standard deviation (SD) were computed for demographics and all drinking outcomes. These were also computed for males and females separately, and potential sex differences were tested by applying the nonparametric Independent Samples Mann–Whitney U test. The effect size for sex differences was computed as *η*^2^ = *Z*^2^/*n* − 1. Differences were considered significant if *P* < 0.05. The analyses were conducted for the overall sample as well age separate age bins: Group 1 (18–25 years old), Group 2 (26–35 years old), Group 3 (36–45 years old), Group 4 (46–55 years old), Group 5 (56–65 years old), Group 6 (66–75 years old) and Group 7 (>75 years old). The Kruskal–Wallis test, including Bonferroni’s correction for multiple comparisons, was used to compare the age groups. The effect size for the Kruskal–Wallis test (*η*^2^) was computed as *η*^2^ = (*H − k* + 1)/(*n − k*), where *H* is the value obtained in the Kruskal–Wallis test, *k* is the number of groups and *n* is the total number of observations. The *η*^2^ ranges from 0 to 1, and its size is commonly interpreted as small (0.01–0.06), moderate (0.06–0.14) or large (≥0.14) (Tomczak and Tomczak, 2014). The interaction between age group and sex was computed using the Aligned Rank Transform test (Wobbrock *et al.*, 2011), and the corresponding effect size was computed as *η_p_*^2^. = (*F*  ^*^  *df*1)/(*F*  ^*^  *df*1 + *df*2) (Friedman, 1982).

To further evaluate the relationship between age and drinking outcomes, Spearman’s rho correlations were computed between age and drinking outcomes. To account for the age-related differences in the amount of alcohol consumed, partial correlations were computed, correcting for eBAC. eBAC was chosen, as in addition to the amount of alcohol consumed, total drinking time, sex and body weight are incorporated in the calculation of eBAC (Watson *et al.*, 1981). Therefore, BAC may be considered as a measure that more comprehensive captures alcohol intake instead of only referring to the number of alcoholic drinks consumed (Verster *et al.*, 2020a). Correlations were considered significant if *P* < 0.05. Using the Fisher *r*-to-*z* transformation (online calculator, available at http://vassarstats.net/rdiff.html), sex differences between the observed correlations were tested (two-tailed). Sex differences were considered significant if *P* < 0.05.

## RESULTS

The dataset comprised of 761 participants with an age range of 18–94 years old (61.6% female). Demographic information is summarized in Table 1.

**Table 1:** Demographics

Demographics	Overall	Men	Women
*N*/%	761/100%	292/38.4%	469/61.6%
Age (years)	42.3 (19.0)	48.0 (19.2)	38.7 (18.0)^^*^^
Height (m)	1.74 (0.09)	1.81 (0.08)	1.70 (0.07)^^*^^
Weight (kg)	77.9 (16.8)	85.5 (14.9)	73.1 (16.2)^^*^^
BMI (kg/m^2^)	25.6 (5.1)	26.0 (4.4)	25.4 (5.4)

Mean and SD (between brackets) are shown. Significant differences between men and women (*P* < 0.05) are indicated by ^*^. Abbreviation: BMI, body mass index.

**Fig. 1.**
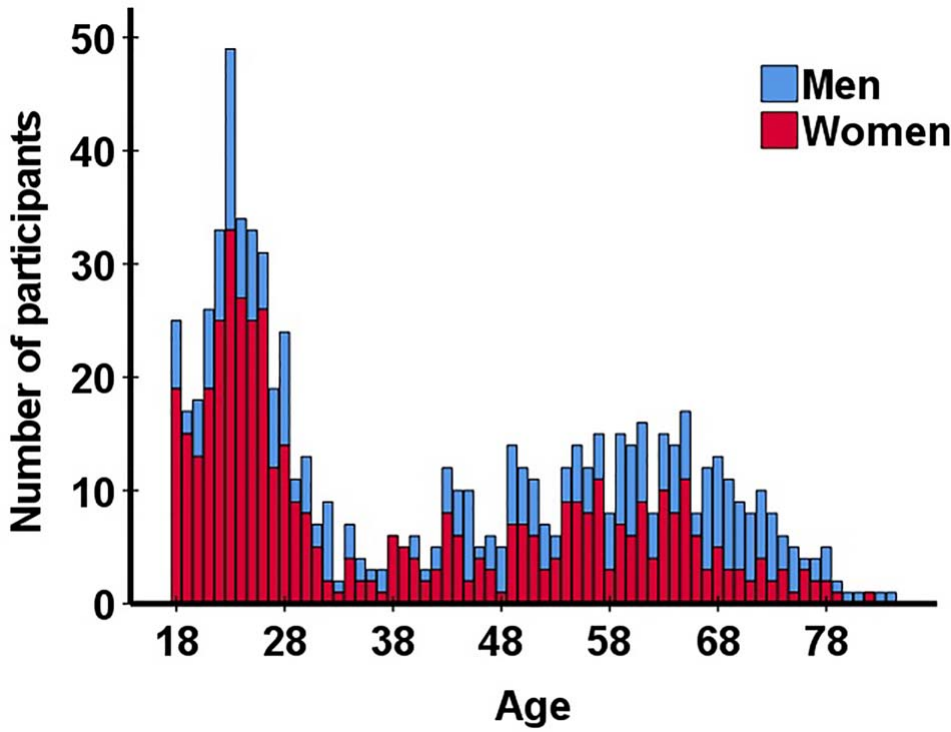
Distribution of the sample according to age and sex.

More women than men participated in the study. This was especially evident among young adults (18–30 year olds). In other age groups, the sexes were more equally represented. The data further show that young adults (18–30 years old) were over-represented in the sample, whereas participants of the age range between 30 and 40 years old least frequently completed the survey. The age distribution of survey participants is summarized in Fig. 1.

**Fig. 2.**
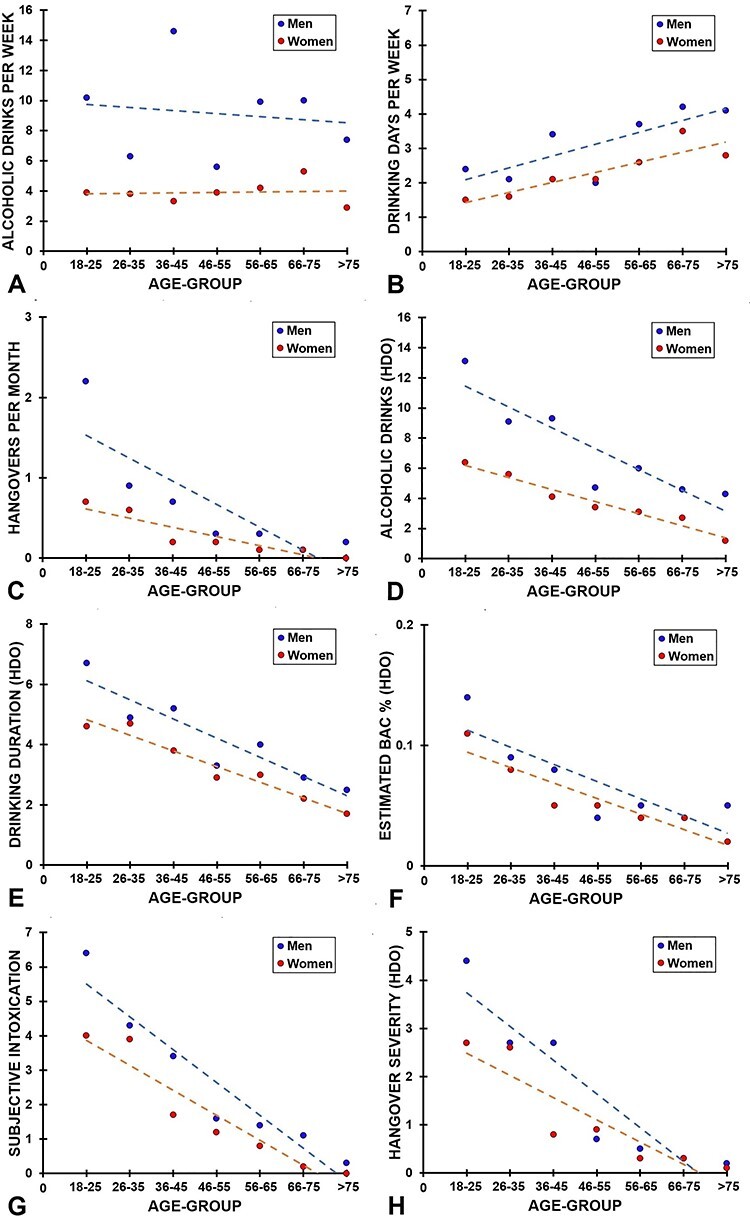
Alcohol consumption outcomes; mean values are presented for each age group, separate for men and women, including linear regression lines; (**A**) number of alcoholic drinks per week, (**B**) drinking days per week, (**C**) hangovers per month, (**D**) number of alcoholic drinks on heaviest drinking occasion, (**E**) drinking duration, (**F**) eBAC, (**G**) subjective intoxication and (**H**) hangover severity; abbreviations: HOD, heaviest drinking occasion.

Figure 2 summarizes the drinking behaviors of the participants. The amount of alcohol consumed per week is summarized in Table 2 and Fig. 2A. In this sample, women consumed significantly less alcohol per week than men (*Z* = −7.96, *P* < 0.0001, *η*^2^ = 0.09), and there was also a significant overall main effect of age group (*H* = 18.27, *P* = 0.006, *η*^2^ = 0.02). The effect of age group for weekly alcohol consumption was significant for men (*H* = 13.85, *P* = 0.031, *η*^2^ = 0.03), but not for women (*H* = 6.47, *P* = 0.373, *η*^2^ = 0.001). The interaction between age group and sex was not significant (*F*_(1,6)_ = 0.00, *P* = 1.00, *η*_p_*^2^ =* 0.00).

**Table 2:** Alcohol consumption per week

Age group (age range)	*N*	Overall	Men	Women
Group 1 (18–25)	219	5.5 (7.0)	10.2 (9.3)	3.9 (5.0)^^*^^
Group 2 (26–35)	120	4.6 (5.5)	6.3 (7.2)	3.8 (4.1)^^*^^
Group 3 (36–45)	54	8.1 (12.8)	14.6 (17.5)	3.3 (3.2)^^*^^
Group 4 (46–55)	88	4.6 (6.2)	5.6 (6.8)^1^	3.9 (5.6)
Group 5 (56–65)	126	6.7 (9.5)	9.9 (12.4)	4.2 (5.4)^^*^^
Group 6 (66–75)	85	8.4 (11.9)^1,4^	10.0 (13.9)	5.3 (5.2)
Group 7 (>75)	19	5.3 (5.6)	7.4 (6.8)	2.9 (2.7)
Overall sample	711	6.0 (8.5)	9.2 (11.4)	3.9 (4.8)^^*^^

Mean and SD (between brackets) are shown. Significant differences between men and women (*P* < 0.05) are indicated by ^*^. Differences (*P* < 0.05) between age groups, after Bonferroni’s correction, are indicated as follows: 1 = significantly different from Group 1, 4 = significantly different from Group 4.

Data on drinking days per week is summarized in Table 3 and Fig. 2B. Men reported significantly more drinking days then women (*Z* = −6.60, *P* < 0.0001, *η*^2^ = 0.06), and there was also a significant overall main effect of age group (*H* = 7.83, *P* < 0.0001, *η*^2^ = 0.09), indicating that with increasing age, the number of drinking days increases. The main effect of age group was significant in both men (*H* = 39.72, *P* < 0.0001, *η*^2^ = 0.12) and women (*H* = 22.74, *P* = 0.001, *η*^2^ = 0.04). The interaction between age group and sex was not significant (*F*_(1,6)_ = 0.41, *P* = 0.875, *η*_p_*^2^ =* 0.41).

**Table 3:** Drinking days per week

Age group (age range)	*N*	Overall	Men	Women
Group 1 (18–25)	219	1.7 (1.3)	2.4 (1.5)	1.5 (1.2)^^*^^
Group 2 (26–35)	120	1.8 (1.3)	2.1 (1.7)	1.6 (1.1)
Group 3 (36–45)	54	2.6 (2.2)	3.4 (2.5)	2.1 (1.8)
Group 4 (46–55)	88	2.1 (2.1)	2.0 (2.2)	2.1 (2.0)
Group 5 (56–65)	126	3.1 (2.3)^1,2,4^	3.7 (2.3)^2,4^	2.6 (2.3)^^*^^
Group 6 (66–75)	85	4.0 (2.5)^1,2,3,4^	4.2 (2.5)^1,2^	3.5 (2.5)^1,2^
Group 7 (>75)	19	3.5 (2.8)	4.1 (2.8)	2.8 (2.8)
Overall sample	711	2.4 (2.1)	3.1 (2.3)	2.0 (1.8)^^*^^

Mean and SD (between brackets) are shown. Significant differences between men and women (*P* < 0.05) are indicated by ^*^. Differences (*P* < 0.05) between age groups, after Bonferroni’s correction, are indicated as follows: 1 = significantly different from Group 1, 2 = significantly different from Group 2, 3 = significantly different from Group 3, 4 = significantly different from Group 4.

The number of hangovers experienced per month is summarized in Table 4 and Fig. 2C. A main effect of age group was found for the number of hangovers experienced per month, indicating that hangovers were more frequently reported by younger age groups (*H* = 127.57, *P* < 0.0001, *η*^2^ = 0.17). The main effect of age group was significant in both men (*H* = 91.54, *P* < 0.0001, *η*^2^ = 0.31) and women (*H* = 69.57, *P* < 0.0001, *η*^2^ = 0.15). Men reported significantly more hangovers than women (*Z* = −2.86, *P* = 0.004, *η*^2^ = 0.01). The interaction between age group and sex was not significant (*F*_(1,6)_ = 1.92, *P* = 0.075, *η*_p_*^2^ =* 0.24).

**Table 4:** Number of hangovers per month

Age group (age range)	*N*	Overall	Men	Women
Group 1 (18–25)	219	1.1 (1.6)	2.2 (2.2)	0.7 (1.1)^^*^^
Group 2 (26–35)	120	0.7 (0.9)	0.9 (1.1)	0.6 (0.8)
Group 3 (36–45)	54	0.4 (0.8)^1^	0.7 (0.7)	0.2 (0.7)^^*^^^,1,2^
Group 4 (46–55)	88	0.3 (0.6)^1,2^	0.3 (0.7)^1,2^	0.2 (0.6)^1,2^
Group 5 (56–65)	126	0.2 (0.7)^1,2^	0.3 (0.9)^1,2^	0.1 (0.4)^^*^^^,1,2^
Group 6 (66–75)	85	0.1 (0.4)^1,2^	0.1 (0.4)^1,2,3^	0.1 (0.4)^1,2^
Group 7 (>75)	19	0.1 (0.3)^1,2^	0.2 (0.3)	0.0 (0.0)
Overall sample	711	0.6 (1.1)	0.8 (1.4)^1^	0.4 (0.9)^^*^^

Mean and SD (between brackets) are shown. Significant differences between men and women (*P* < 0.05) are indicated by ^*^. Differences (*P* < 0.05) between age groups, after Bonferroni’s correction, are indicated as follows: 1 = significantly different from Group 1, 2 = significantly different from Group 2, 3 = significantly different from Group 3, 4 = significantly different from Group 4.

Table 5 and Fig. 2E summarize the number of alcoholic drinks consumed on the heaviest drinking occasion. A significant age group effect was found (*H* = 85.19, *P* < 0.0001, *η*^2^ = 0.11), indicating that younger age groups consumed significantly more alcohol than older age groups. The effect of age group was significant for both men (*H* = 57.93, *P* < 0.0001, *η*^2^ = 0.19) and women (*H* = 66.50, *P* < 0.0001, *η*^2^ = 0.14). Also, men consumed significantly more alcohol than women (*Z* = −5.08, *P* < 0.0001, *η*^2^ = 0.04). The interaction between age group and sex was not significant (*F*_(1,6)_ = 0.72, *P* = 0.638, *η*_p_*^2^ =* 0.08).

**Table 5:** Number of alcoholic drinks on heaviest drinking occasion

Age group (age range)	*N*	Overall	Men	Women
Group 1 (18–25)	219	8.2 (6.5)	13.1 (7.8)	6.4 (4.9)^^*^^
Group 2 (26–35)	120	6.8 (6.2)	9.1 (8.5)	5.6 (4.1)
Group 3 (36–45)	54	6.3 (6.3)	9.3 (8.3)	4.1 (2.6)^^*^^
Group 4 (46–55)	88	4.0 (3.5)^1,2^	4.7 (4.0)^1^	3.4 (3.0)^1,2^
Group 5 (56–65)	126	4.3 (4.2)^1,2^	6.0 (5.4)^1^	3.1 (2.5)^^*^^^,1,2^
Group 6 (66–75)	85	3.9 (3.5)^1,2^	4.6 (4.0)^1^	2.7 (1.7)^^*^^^,1,2^
Group 7 (>75)	19	2.8 (2.5)^1,2^	4.3 (2.5)^1^	1.2 (0.8)^^*^^^,1,2^
Overall sample	711	6.0 (5.7)	7.7 (7.1)	4.8 (4.2)^^*^^

Mean and SD (between brackets) are shown. Significant differences between men and women (*P* < 0.05) are indicated by ^*^. Differences (*P* < 0.05) between age groups, after Bonferroni’s correction, are indicated as follows: 1 = significantly different from Group 1, 2 = significantly different from Group 2, 3 = significantly different from Group 3, 4 = significantly different from Group 4.

Table 6 and Fig. 2E summarize the hours of drinking on the heaviest drinking occasion. The significant main effect of age group (*H* = 101.21, *P* < 0.0001, *η*^2^ = 0.14) indicates that the drinking duration of the younger age groups was significantly longer compared to the older age groups. The effect of age group was significant for both men (*H* = 58.10, *P* < 0.0001, *η*^2^ = 0.19) and women (*H* = 61.04, *P* < 0.0001, *η*^2^ = 0.13). No significant differences between men and women were observed (*Z* = −134, *P* = 0.180, *η*  ^2^ = 0.002). The interaction between age group and sex was also not significant (*F*_(1,6)_ = 0.19, *P* = 0.981, *η*_p_*^2^ =* 0.03).

**Table 6:** Drinking duration (h) on heaviest drinking occasion

Age group (age range)	*N*	Overall	Men	Women
Group 1 (18–25)	219	5.1 (2.8)	6.7 (3.2)	4.6 (2.4)^^*^^
Group 2 (26–35)	120	4.8 (2.9)	4.9 (3.3)	4.7 (2.7)
Group 3 (36–45)	54	4.4 (3.1)	5.2 (3.3)	3.8 (2.8)
Group 4 (46–55)	88	3.1 (2.6)^1,2^	3.3 (3.0)^1^	2.9 (2.2)^1,2^
Group 5 (56–65)	126	3.4 (2.9)^1,2^	4.0 (3.6)^1^	3.0 (2.2)^1,2^
Group 6 (66–75)	85	2.7 (1.9)^1,2,3^	2.9 (2.1)^1,2^	2.2 (1.4)^1,2^
Group 7 (>75)	19	2.1 (1.8)^1,2,3^	2.5 (1.7)^1^	1.7 (1.8)^1,2^
Overall sample	711	4.1 (2.9)	4.4 (3.3)	3.9 (2.5)

Mean and SD (between brackets) are shown. Significant differences between men and women (*P* < 0.05) are indicated by ^*^. Differences (*P* < 0.05) between age groups, after Bonferroni’s correction, are indicated as follows: 1 = significantly different from Group 1, 2 = significantly different from Group 2, 3 = significantly different from Group 3, 4 = significantly different from Group 4.

Table 7 and Fig. 2F summarize the eBAC on the heaviest drinking occasion. The main effect of age group (*H* = 95.22, *P* < 0.0001, *η*^2^ = 0.13) indicates that the eBAC of the younger age groups was significantly higher compared to the older age groups. The effect of age group was significant in both men (*H* = 48.84, *P* < 0.0001, *η*^2^ = 0.16) and women (*H* = 53.54, *P* < 0.0001, *η*^2^ = 0.11). No significant sex effect was found (*Z* = 0.458, *P* = 0.647, *η*  ^2^ = 0.0003). Also, the interaction between age group and sex was not significant (*F*_(1,6)_ = 1.03, *P* = 0.404, *η*_p_*^2^ =* 0.15).

**Table 7:** eBAC (%) on heaviest drinking occasion

Age group (age range)	*N*	Overall	Men	Women
Group 1 (18–25)	219	0.12 (0.10)	0.14 (0.10)	0.11 (0.10)^^*^^
Group 2 (26–35)	120	0.08 (0.09)^1^	0.09 (0.11)	0.08 (0.07)
Group 3 (36–45)	54	0.06 (0.08)^1^	0.08 (0.11)^1^	0.05 (0.04)
Group 4 (46–55)	88	0.05 (0.05)^1^	0.04 (0.04)^1^	0.05 (0.05)^1^
Group 5 (56–65)	126	0.04 (0.05)^1,2^	0.05 (0.06)^1^	0.04 (0.04)^1,2^
Group 6 (66–75)	85	0.04 (0.04)^1,2^	0.04 (0.05)^1^	0.04 (0.04)^1^
Group 7 (>75)	19	0.04 (0.04)^1^	0.05 (0.05)	0.02 (0.02)
Overall sample	711	0.07 (0.09)	0.08 (0.09)	0.07 (0.08)

Mean and SD (between brackets) are shown. Significant differences between men and women (*P* < 0.05) are indicated by ^*^. Differences (*P* < 0.05) between age groups, after Bonferroni’s correction, are indicated as follows: 1 = significantly different from Group 1, 2 = significantly different from Group 2, 3 = significantly different from Group 3, 4 = significantly different from Group 4.

Table 8 and Fig. 2G summarize the subjective intoxication ratings on the heaviest drinking occasion. The significant main effect of age group (*H* = 216.05, *P* < 0.0001, *η*^2^ = 0.30) indicates that subjective intoxication ratings of the younger age groups were significantly higher compared to the older age groups. The main effect of age group was significant for both men (*H* = 112.33, *P* < 0.0001, *η*^2^ = 0.39) and women (*H* = 130.13, *P* < 0.0001, *η*^2^ = 0.29). No significant overall sex effect was found (*Z* = −1.34, *P* = 0.182, *η*^2^ = 0.003). The significant interaction between age group and sex (*F*_(1,6)_ = 12.37, *P* < 0.0001, *η*_p_*^2^ =* 0.67) indicates that the difference in reported subjective intoxication between men and women was greatest in the younger age groups but became significantly smaller in the older age groups.

**Table 8:** Subjective intoxication on heaviest drinking occasion

Age group (age range)	*N*	Overall	Men	Women
Group 1 (18–25)	219	4.6 (3.2)	6.4 (3.0)	4.0 (3.0)^^*^^
Group 2 (26–35)	120	4.1 (3.0)	4.3 (2.9)	3.9 (3.0)
Group 3 (36–45)	54	2.5 (2.9)^1,2^	3.4 (3.2)^1^	1.7 (2.6)^^*^^^,1,2^
Group 4 (46–55)	88	1.4 (2.1)^1,2^	1.6 (2.3)^1,2^	1.2 (1.9)^1,2^
Group 5 (56–65)	126	1.1 (1.8)^1,2^	1.4 (2.0)^1,2^	0.8 (1.6)^^*^^^,1,2^
Group 6 (66–75)	85	0.8 (1.8)^1,2,3^	1.1 (2.1)^1,2,3^	0.2 (0.8)^^*^^^,1,2^
Group 7 (>75)	19	0.2 (0.4)^1,2^	0.3 (0.5)^1,2^	0.0 (0.0)^1,2^
Overall sample	711	2.8 (3.1)	3.0 (3.3)	2.6 (3.0)

Mean and SD (between brackets) are shown. Significant differences between men and women (*P* < 0.05) are indicated by ^*^. Differences (*P* < 0.05) between age groups, after Bonferroni’s correction, are indicated as follows: 1 = significantly different from Group 1, 2 = significantly different from Group 2, 3 = significantly different from Group 3, 4 = significantly different from Group 4.

Table 9 and Fig. 2H summarize the reported hangover severity experienced after their heaviest drinking occasion. The significant main effect of age group (*H* = 167.17, *P* < 0.0001, *η*^2^ = 0.23) indicates that the hangover severity of the younger age groups was significantly higher compared to the older age groups. The effect of age group was significant in both men (*H* = 103.73, *P* < 0.0001, *η*^2^ = 0.36) and women (*H* = 90.77, *P* < 0.0001, *η*^2^ = 0.20). The main effect of sex was not significant (*Z* = −0.53, *P* = 0.597, *η*^2^ = 0.0004). The significant interaction between age group and sex (*F*_(1,6)_ = 7.32, *P* < 0.0001, *η*_p_*^2^ =* 0.55) indicates that the differences in hangover severity between men and women were greatest in the younger age groups but became significantly smaller or absent in the older age groups.

**Table 9:** Hangover severity on heaviest drinking occasion

Age group (age range)	*N*	Overall	Men	Women
Group 1 (18–25)	219	3.1 (3.2)	4.4 (3.2)	2.7 (3.2)^^*^^
Group 2 (26–35)	120	2.6 (2.7)	2.7 (2.8)	2.6 (2.7)
Group 3 (36–45)	54	1.6 (2.5)^1^	2.7 (2.9)	0.8 (1.9)^^*^^^,1,2^
Group 4 (46–55)	88	0.8 (1.8)^1,2^	0.7 (1.5)^1,2^	0.9 (2.0)^1,2^
Group 5 (56–65)	126	0.4 (1.1)^1,2,3^	0.5 (1.3)^1,2,3^	0.3 (0.9)^1,2^
Group 6 (66–75)	85	0.2 (0.9)^1,2,3^	0.3 (1.1)^1,2,3^	0.3 (0.2)^1,2^
Group 7 (>75)	19	0.2 (0.4)^1,2^	0.2 (0.4)^1^	0.1 (0.3)^2^
Overall sample	711	1.7 (2.8)	1.8 (2.7)	1.7 (2.7)

Mean and SD (between brackets) are shown. Significant differences between men and women (*P* < 0.05) are indicated by ^*^. Differences (*P* < 0.05) between age groups, after Bonferroni’s correction, are indicated as follows: 1 = significantly different from Group 1, 2 = significantly different from Group 2, 3 = significantly different from Group 3, 4 = significantly different from Group 4.

Table 10 summarizes the Spearman’s correlations between age and the drinking outcomes. It is evident from Table 10 that with increasing age, participants consume more alcohol per day and report more drinking days per week. Although the correlation between the amount of alcohol consumed per week and age is positive and significant, the actual increase in weekly alcohol consumption with increasing age is only modest. However, the positive correlation between alcohol consumption and drinking days per week (see Table 10) does suggest that with increasing age, alcohol consumption is spread over more days per week.

**Table 10:** Correlations between alcohol consumption outcomes and age

Alcohol consumption	Overall	Men	Women
Assessed over a 2-month period			
Alcoholic drinks/week	*r* = 0.085, *P* = 0.023^*^	*r* = −0.026, *P* = 0.660	*r* = 0.049, *P* = 0.312
Drinking days/week	*r* = 0.281, *P* < 0.000^*^	*r* = 0.271, *P* < 0.000^*^	*r* = 0.231, *P* < 0.000^*^
Hangovers per month	*r* = −0.391, *P* < 0.000^*^	*r* = −0.549, *P* < 0.000^*^	*r* = −0.332, *P* < 0.000^*^^,†^
Heaviest drinking occasion			
Number of drinks	*r* = −0.327, *P* < 0.000^*^	*r* = −0.408, *P* < 0.000^*^	*r* = −0.365, *P* < 0.000^*^
Drinking duration (h)	*r* = −0.360, *P* < 0.000^*^	*r* = −0.424, *P* < 0.000^*^	*r* = −0.336, *P* < 0.000^*^
eBAC (%)	*r* = −0.358, *P* < 0.000^*^	*r* = −0.367, *P* < 0.000^*^	*r* = −0.347, *P* < 0.000^*^
Subjective intoxication	*r* = −0.520, *P* < 0.000^*^	*r* = −0.608, *P* < 0.000^*^	*r* = −0.491, *P* < 0.000^*^^,†^
Hangover severity	*r* = −0.446, *P* < 0.000^*^	*r* = −0.569, *P* < 0.000^*^	*r* = −0.375, *P* < 0.000^*^^,†^

Spearman’s rho correlation coefficients (*r*) and corresponding *P*-values are shown. Significant correlations (*P* < 0.05) are indicated by ^*^. Significant sex differences for the correlations (*P* < 0.05) are indicated by ^†^.

The observed correlations between age and drinking outcomes for the heaviest drinking occasion were stronger in men compared to women, and the difference between the correlations was significant for subjective intoxication (*z* = −2.25, *P* = 0.024) and hangover severity (*z* = −3.36, *P* = 0.0008). Also, the correlation between age and hangover frequency was significantly stronger in men than women (*z* = −3.63, *P* = 0.0003).

For the heaviest drinking occasion, significantly fewer alcoholic drinks were reported alongside a significantly shorter drinking time as age increased. As shown by the negative correlations in Table 10, this results in a significant lower eBAC with increasing age. Subsequently, as shown by the negative correlations in Table 10, with increasing age, corresponding subjective intoxication ratings and next-day hangover severity scores are lower. In addition, significantly fewer hangovers per month are reported with increasing age.

**Fig. 3.**
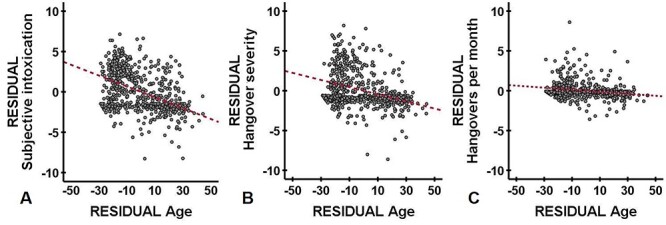
Partial correlations between age and drinking outcomes, correcting for eBAC. residual regression plots are shown; the partial correlations are corrected for eBAC; age was negatively and significantly correlated with (**A**) subjective intoxication (*r* = −0.444, *P* < 0.0001), (**B**) hangover severity (*r* = −0.327, *P* < 0.0001) and (**C**) hangover frequency (*r* = −0.195, *P* < 0.0001). The red line represents the regression line.

As frequency and severity of hangovers depend on the amount and the timeframe of alcohol consumption, partial correlations, correcting for eBAC, were computed to further investigate the relationship between age and subjective intoxication, hangover frequency and severity. First, the analysis revealed a significant and negative partial correlation between age and subjective intoxication (*r* = −0.444, *P* < 0.0001), indicating that with increasing age, drinkers report significantly lower levels of intoxication (see Fig. 3A). This observation suggests that with increasing age (and alcohol use), tolerance develops to alcohol’s acute effects. Second, the analysis revealed a significant and negative partial correlation between age and hangover severity (*r* = −0.327, *P* < 0.0001), indicating that with increasing age, less severe hangovers are experienced (see Fig. 3B). This observation suggests that with increasing age, tolerance develops to the severity of experienced hangover symptoms. Third, the analysis revealed a significant and negative partial correlation between age and hangover frequency (*r* = −0.195, *P* < 0.0001), indicating that with increasing age, fewer hangovers are experienced (see Fig. 3C).

Conducting the same partial correlations, but correcting for the amount of ethanol consumed instead of eBAC, yielded comparable significant and negative partial correlations between age and subjective intoxication (*r* = −0.464, *P* < 0.0001), age and hangover severity (*r* = −0.341, *P* < 0.0001) and between age and hangover frequency (*r* = −0.198, *P* < 0.0001).

Finally, previous research has shown subjective intoxication to be the strongest predictor of next-day hangover severity (Verster *et al.*, 2020a). Indeed, when correcting for both eBAC and subjective intoxication, the correlations between age and hangover severity (*r* = −0.048, *P* = 0.205) and between age and hangover frequency (*r* = 0.012, *P* = 0.753) were no longer significant. Also in line with previous research (Verster *et al.*, 2019), the correlation between hangover frequency and severity was highly significant (*r* = 0.710, *P* < 0.0001), suggesting reverse tolerance.

## DISCUSSION

Alcohol and hangover research are usually conducted in young adult students, and thus a limited age range is investigated (e.g. 18–30 years old). This study shows that it is important to also investigate alcohol hangover in older age groups. The results suggest that with increasing age, tolerance develops for both subjective intoxication and the after-effects of alcohol consumption. That is, after controlling for eBAC, significant negative correlations were found between age and subjective intoxication, age and hangover severity and age and hangover frequency (see Fig. 3). These findings are in line with previous research showing that with increasing age, hangover frequency declines (Piasecki *et al.*, 2005; Tolstrup *et al.*, 2014). The current study suggests that this finding extends to hangover severity.

Possible explanations for reduced hangover severity with age may be related to (a) the observed reduction in subjective intoxication when growing older and (b) a gradual decline in pain sensitivity with aging.

### Reduced subjective intoxication when aging

Although the relationship between age and subjective intoxication has received little research attention, the available data on subjective intoxication and aging support our findings. Jones and Neri (1985) examined alcohol metabolism and subjective intoxication in 48 men after an alcohol challenge (0.68 g/kg). In this relatively small study, participants were allocated to one of four different age groups (*N* = 12 per group), 20–29, 30–39, 40–49 or 50–59 years old. The authors reported significant positive correlations between age and BAC (*r* = 0.35, *P* < 0.05) and between age and subjective intoxication (*r* = 0.45, *P* < 0.01). Lower subjective intoxication rates were found among 20–29 years old, whereas the other age groups (30–59 years old) had higher ratings and did not significantly differ from each other. Although these findings are opposite to those in the current study, there are potential methodological explanations for this. First, the study was designed to test three participants simultaneously. The authors state that, with simultaneous testing, it is likely that drinkers may have exchanged and discussed their opinions about feelings of intoxication and that it could be that, in this social context, subjective intoxication ratings among younger drinkers were trivialized to convince other participants that they were more resistant to alcohol effects. Of more importance, in the presented statistical analysis, the relationship between age and subjective intoxication did not control for BAC. This limits the interpretation of the observed positive correlation, as the authors also found that increasing BACs resulted in higher intoxication ratings. Not correcting for the amount of alcohol consumed (i.e. BAC) could explain why the observed association between age and subjective intoxication had a positive relationship. A closer look at the data (Figure 3 in their paper) supports this notion. If one does not consider the 20–29-year-old group but only looks at the 30–59-year-old participants, the direction of the association between age and subjective intoxication is negative, which is similar to what is observed in our study.

A negative relationship between age and self-reported drunkenness was confirmed by Neil and Martin (1989) in a study examining drunkenness in *N* = 412 Australian twins (*N* = 213 women and *N* = 199 men). In an experimental session, participants consumed 0.75 g/kg alcohol, and subjective intoxication was assessed 1–3 h after drinking using a scale ranging from 0 (quite sober) to 10 (the most drunk I have ever been). The authors reported that drunkenness scores decreased with increasing age. Although drunkenness ratings were significantly higher in women than men, sex had no impact on showing a negative relationship between age and drunkenness. It was concluded that older persons report less drunkenness than younger persons and that this observation may be related to the development of tolerance (i.e. that higher levels of alcohol are needed by older persons to feel the same alcohol effects).

More recently, Kaestle *et al.* (2018) examined objective intoxication (using a breathalyzer test) and subjective intoxication in a large on-premise study among *N* = 4628 participants. A random sample of alcohol consumers was interviewed in night-time entertainment districts on Friday and Saturday nights in five Australian cities. Interviews were conducted between 10 pm and 3 am and lasted approximately for 3–15 min. Subjective intoxication was rated on a 0–10 scale, and actual BAC was assessed with a breathalyzer. For the analysis, age was categorized as (a) 18–20 years old or (b) >20 years old. This study confirmed that being young was associated with higher reported levels of self-assessed intoxication when compared to older people at the same BAC level.

Finally, the role of (reverse) tolerance as explanatory factor should be further investigated, as it has been shown that experiencing hangovers more frequently was associated with having more severe hangovers (Verster *et al.*, 2019). In the current study, hangovers were experienced more frequently in young individuals compared to older individuals. At the same time, the correlation between hangover frequency and severity was highly significant.

Taken together, these studies suggest that with increasing age, at the same BAC level, a decline in reported subjective intoxication is seen. As subjective intoxication is one of the most important predictors of hangover severity (Verster *et al.*, 2020a), this may also explain the negative correlation between age and hangover severity and age and hangover frequency.

### The impact of sensitivity to pain on experiencing hangovers

An explanation for our findings may be related to recent research by Royle *et al.* (2020) who investigated the relationship between hangover severity and pain catastrophizing. Individuals with increased levels of pain catastrophizing are excessively oriented at the negative cognitive and emotional aspects of pain, i.e. an exaggerated response to pain, characterized by worry, fear and difficulty in directing attention away from pain (Turk and Rudy, 1992), and they report greater pain intensity (Sullivan and Neish, 1998). Royle *et al.* (2020) reported a significant positive relationship between hangover severity and pain catastrophizing. In particular, people who scored higher on rumination, i.e. drinkers with a higher focus on symptoms of distress, reported more severe hangovers. Many factors modulate one’s level of pain catastrophizing, including personality. In this context, it has also been suggested that the level of pain catastrophizing is related to age (Ruscheweyh *et al.*, 2011). According to the lifespan theory of emotion, it was hypothesized that, with increasing age, individuals possess greater social and cognitive resources and become more effective in anticipating negative emotions (Ruscheweyh *et al.*, 2011). Alternatively, age-related social norms for the expression of distress may have an impact, such as older individuals having a more stoic orientation toward the expression of distress (Sullivan and Neish, 1998). Further, age-related changes in central nervous system (CNS) functioning associated with the loss and reduced functioning of neurons, dendrites and synapses and reduced levels of neurotransmitters may affect the processing of pain stimuli (Sullivan and Neish, 1998). However, in contrast to these hypotheses, a recent meta-analysis, including all available literature, revealed that pain catastrophizing does not differ according to sex or age (Wheeler *et al.*, 2019).

Another recent meta-analysis found that pain sensitivity declines with increasing age (El Tumi *et al.*, 2017). In other words, reported pain intensity ratings (for the same pain stimulus) are greater in younger adults than those reported by older adults. It thus might be that younger drinkers tend to overestimate the magnitude of subjective intoxication and hangover symptom severity, whereas older adults, who are more experienced with drunkenness and hangovers, have a more modest judgment of the magnitude of these effects. This corresponds to the observation that, after correcting for the amount of alcohol consumed, the hangover severity ratings of younger drinkers are greater than those of older drinkers, and it explains the negative correlations between age and hangover severity and age and hangover frequency.

### Limitations and directions for future research

A limitation of the current study comprises the fact that data were recalled retrospectively. This may have introduced recall bias or inaccurate reporting. However, there is no reason to assume that this would have affected younger participants differently than older participants. As such, we feel that possible recall bias would not have affected the observed associations with age. Nevertheless, future studies with a prospective study design including real-time alcohol consumption recordings should confirm our findings. Including objective BAC measurements in such a study would provide further insight into the assessments of subjective intoxication. Future studies should also address whether age differentially affects the cognitive impairments associated with alcohol hangover and its functional consequences, which are well documented for the younger age group but not for older adults (Prat *et al.*, 2008; Prat *et al.*, 2009; Gunn *et al.*, 2018).

Second, hangover severity was assessed using a single-item scale. Although this may be considered a limitation, recent research shows the opposite. Recent research confirms that single-item assessments are equally capable of assessing a construct than multiple-item scales (Verster *et al.*, 2021). Moreover, the use of single-item assessments may be even preferred over multiple-item assessments as they incorporate assessments of all aspects of alcohol hangover (including the presence, severity and impact of the construct under investigation) compared to the scale that comprises composite symptom scores (Verster *et al.*, 2020c, 2021). Third, the observed relationship between hangover severity and age may be influenced by other factors such as physical state. Future studies should investigate the impact of demographic and health characteristics of drinkers on the relationship between age and hangover severity. Finally, the current study demonstrates that it is important to take age into account when investigating alcohol hangover. Future studies should therefore not limit recruitment to student or young adult samples but should investigate a more representative sample of the population as a whole, including older participants.

## CONCLUSIONS

Taken together, our study confirms that both subjective intoxication and hangover frequency decline with age. The study also adds that hangover severity declines with age, and this relationship remains after controlling for eBAC or the amount of alcohol consumed.

Significant interactions between age group and sex were found for both subjective intoxication and hangover severity, indicating that the sex differences in these variables were greatest in the younger age groups but became significantly smaller or absent in the older age groups. The relationship between age and hangover severity is strongly mediated by subjective intoxication. The analysis revealed that, when controlling for subjective intoxication level, the correlations between age and hangover severity and between age and hangover frequency were no longer statistically significant. This observation is in line with literature showing a decline in subjective intoxication with aging and supports previous findings that subjective intoxication is an important predictor of hangover severity. Finally, an age-related decline in pain sensitivity to pain may in part explain the observed negative relationship between aging and the frequency and severity of alcohol hangovers.

## Authors’ contributions

N.R.S., A.S.M.S., H.M.S., S.B., A.S. and J.C.V. contributed to conceptualization, design and methodology of the study; investigation was done by H.M.S.; J.C.V. and G.B. conducted the statistical analysis and J.C.V. prepared the original draft; all authors critically reviewed the paper and approved the final version.

## Funding

This research received no external funding.

## Conflict of interest statement

Over the past 3 years, J.C.V. has received grants/research support from Janssen Research and Development and Sequential Medicine and has acted as a consultant/advisor for More Labs, Red Bull, Sen-Jam Pharmaceutical, Toast!, Tomo and ZBiotics. S.B. has received funding from Red Bull GmbH, Kemin Foods, Sanofi Aventis, Phoenix Pharmaceutical and GlaxoSmithKline. Over the past 36 months, A.S. has held research grants from Abbott Nutrition, Arla Foods, Bayer, BioRevive, DuPont, Kemin Foods, Nestlé, Nutricia-Danone and Verdure Sciences. He has acted as a consultant/expert advisor to Arepa Nootroptics, Bayer, Coca-Cola, Danone, Naturex, Nestlé, Pfizer, Sanofi and Sen-Jam Pharmaceutical and has received travel/hospitality/speaker fees from Bayer, Sanofi and Verdure Sciences. The other authors have no potential conflicts of interest to disclose.

## Data availability

The data are available from the corresponding author upon reasonable request.
