# Corrigendum to: Alcohol Hangover Across the Lifespan: Impact Of Sex and Age

**DOI:** 10.1093/alcalc/agab037

**Published:** 2021-05-07

**Authors:** Joris C Verster, Noortje R Severeijns, Annabel S M Sips, Hama M Saeed, Sarah Benson, Andrew Scholey, Gillian Bruce

**Affiliations:** 1 Division of Pharmacology, Utrecht Institute for Pharmaceutical Sciences (UIPS), Utrecht University, Utrecht 3584 CG, The Netherlands; 2 Centre for Human Psychopharmacology, Swinburne University of Technology, Melbourne VIC 3122, Australia; 3 Division of Psychology and Social Work, School of Education and Social Sciences, University of the West of Scotland, Paisley PA1 2BE, UK

In the originally published version of this paper, figure 3 was missing a panel. This has now been corrected online.

